# Symmetry breaking in the female germline cyst

**DOI:** 10.1126/science.abj3125

**Published:** 2021-11-11

**Authors:** D. Nashchekin, L. Busby, M. Jakobs, I. Squires, D. Johnston

**Affiliations:** 1 The Gurdon Institute and the Department of Genetics, University of Cambridge; Tennis Court Road, Cambridge CB2 1QN, United Kingdom; 2 The Department of Physiology, Development and Neuroscience, University of Cambridge; Cambridge CB2 3DY, United Kingdom

## Abstract

In mammals and flies, only one cell in a multicellular female germline cyst becomes an oocyte, but how symmetry is broken to select the oocyte is unknown. Here we show that the microtubule minus end-stabilizing protein, Patronin/CAMSAP marks the future *Drosophila* oocyte and is required for oocyte specification. The spectraplakin, Shot, recruits Patronin to the fusome, a branched structure extending into all cyst cells. Patronin stabilizes more microtubules in the cell with most fusome. Our data suggest that this weak asymmetry is amplified by Dynein-dependent transport of Patronin-stabilized microtubules. This forms a polarized microtubule network, along which Dynein transports oocyte determinants into the presumptive oocyte. Thus, Patronin amplifies a weak fusome anisotropy to break symmetry and select one cell to become the oocyte.

In many organisms, not all female germ cells develop into oocytes. Some cells become accessory cells that contribute material to the oocyte (1). Mouse female germ cells form cysts of up to 30 cells, but most cells undergo apoptosis after transferring cytoplasm and centrosomes to the small number of cells that become oocytes (2, 3). In *Drosophila*, germline cyst formation starts in the germarium, which has 3 regions. A stem cell produces a cystoblast, which then divides four times with incomplete cytokinesis to generate a cyst of 16 germ cells connected by intercellular bridges, “ring canals” (4, 5). As the cyst moves through regions 2a-b of the germarium, it is surrounded by epithelial follicle cells and then rounds up in region 3 to form a follicle. By this stage, one cell has been selected as the oocyte, whereas others become nurse cells (Fig. 1A). Oocyte selection depends on the formation of a noncentrosomal microtubule organizing center (ncMTOC) in the future oocyte that organizes a polarized microtubule network that directs the dynein-dependent transport of cell fate determinants and centrosomes into the pro-oocyte (6–8) (Fig. 1A). How symmetry is broken to specify which cell contains the ncMTOC and becomes the oocyte is unclear.

Patronin and its vertebrate orthologues (CAMSAPs) are microtubule minus end binding proteins that have been recently found to be essential components of ncMTOCs (9–13). To investigate the role of Patronin in oocyte determination, we examined the distribution of oocyte markers in *patronin^c9-c5^* mutant cysts (Fig. 1B-C and S1). In wild-type cysts, Orb and centrosomes accumulate in future oocytes in regions 2b-3 (14–16), but they are rarely localized in *patronin* mutants (24% and 3% of mutant cysts respectively) (Fig. 1B-C). Several germ cells enter meiosis in region 2a and accumulate the synaptonemal complex protein C(3)G. C(3)G becomes restricted to two cells in region 2b and to the oocyte in region 3 (17) (Fig. S1). C(3)G is not localized in region 3 of *patronin* cysts and 44% of the cysts in region 2b have 3 cells in meiosis (Fig. S1). Thus, Patronin is required for oocyte determination.

To examine whether Patronin is asymmetrically distributed in the cyst, we imaged germaria expressing endogenously tagged Patronin-Kate. Patronin starts to accumulate in a single cell in each cyst in region 2a, earlier than other markers for the presumptive oocyte, and remains in one cell in regions 2b-3, where it forms distinct foci in the cytoplasm (Fig. 2A-2A’). This cell will become the oocyte, as it is also labelled by Orb (Fig. 2B) and C(3)G (Fig. 2C). *patronin* mRNA is not localized within the cyst and Patronin expressed from a cDNA with heterologous UTRs and promoter shows a similar distribution to the endogenous protein, indicating that Patronin is localized as a protein and not through transcription in this cell or mRNA localization (Fig. 2B-C and Fig. S2).

Dynein does not localize to the presumptive oocyte in *patronin* mutant cysts (Fig. 3A). This suggests that the loss of Patronin disrupts the formation of the MTOC in the pro-oocyte, leading to loss of the polarized microtubule network along which Dynein transports cargoes into one cell. As most of MT plus ends accumulate at the site of MT nucleation, we used the MT plus end-tracking protein EB1-GFP to visualize the putative MTOC in the cyst. The majority of EB1-GFP comets localize to one cell in regions 2b-3 (Fig. 3B-C, Movies S1-S2). Moreover, the densest EB1-GFP signal co-localizes with the Patronin foci in the same cell, suggesting that the latter are the MTOCs formed in the pro-oocyte (Fig. 3D). This asymmetric distribution of EB1-GFP is lost in *patronin* cysts, where EB1-GFP comets are distributed more homogeneously (Fig. 3B-C, Movies S3-S4). Patronin is therefore required for MTOC formation in the presumptive oocyte and the organization of a polarized MT network.

Wild-type cysts contain a population of stable, acetylated MTs that form along the fusome, an ER, spectrin, and actin-rich structure that connects all cells of the cyst (16–19) (Fig. S3). In *patronin* mutant cysts, there is a 2.5 fold reduction in stable MTs (Fig. 3E and S3). Thus, in the absence of Patronin, the whole organization of MTs in the cyst is disrupted. Patronin binds MT minus ends and stabilizes MTs by protecting theirs minus ends against kinesin-13 induced depolymerization (11, 13). Our results suggest that early accumulation of Patronin in only one cell of the cyst stabilizes MT minus ends there, leading to dynein-dependent transport into this cell, the formation of MTOCs and the subsequent specification of the oocyte.

To examine whether centrosomes contribute to the formation of Patronin MTOCs, we imaged cysts expressing endogenously tagged Patronin-YFP and the centrosomal protein Asterless-Cherry. Although centrosomal clusters localize near Patronin foci, the Asterless and Patronin signals only partially overlap and most Patronin foci lie outside the centrosomal cluster, indicating that Patronin MTOCs are noncentrosomal (Fig. S4A). Centrosomes have been proposed to be inactive during their migration into the oocyte, and they lack crucial components of the PCM (8). To test whether centrosomes contribute to microtubule organization, we imaged cysts expressing EB1-GFP and Asterless-Cherry. The centrosomes show strong MT nucleating activity in region 1, where they organize the mitotic spindles (Fig. S4B and Movie S5). However, only some Asterless-Cherry labelled centrosomes in the presumptive oocyte produce EB1-GFP comets in region 2b (Fig. S4C and Movie S6). Thus, Patronin-dependent ncMTOCs create the initial asymmetry in MT organization that leads to the accumulation of centrosomes in the pro-oocyte, which may then be amplified by activation of some centrosomes in this cell. The close proximity of the active centrosomes to the ncMTOCs, raises the possibility that new MTs produced by these centrosomes are released and then captured and stabilized by Patronin in ncMTOCs, a mechanism described for CAMSAP proteins (20).

The observation that Patronin is the earliest known marker for the future oocyte raises the question of how symmetry is broken in the cyst to enrich Patronin in one cell. One proposed mechanism for symmetry-breaking is that the cell that inherits the most fusome becomes the presumptive oocyte (21). The fusome is asymmetrically partitioned during the mitoses in region 1, so that mother cells inherit more material than their daughters and one of the two cells with four ring canals has more fusome than the rest (19). To examine whether Patronin associates with the fusome, we imaged germaria expressing endogenously-tagged Patronin-YFP and the fusome marker, Hts-Cherry. Patronin localizes on the fusome in early region 2a, but becomes concentrated in one cell as the cyst progresses towards region 3 (Fig. 4A and S5A). When the MTs are depolymerized with colcemid, however, Patronin remains on the fusome in regions 2b-3 (Fig. 4B). Thus, the fusome determines the initial localization of Patronin in early region 2a, including its slight enrichment in the pro-oocyte, which is then amplified by a MT-dependent process.

The spectraplakin Shot, localizes to the fusome, is required for the oocyte specification, and recruits Patronin to ncMTOCs in the oocyte later in oogenesis, making it a good candidate for a factor that links Patronin to the fusome (13, 17). In *shot* ^-^ cysts, Patronin does not accumulate in one cell and fails to form foci (Fig. 4C). Furthermore, loss of Shot prevents Patronin from associating with the fusome (Fig. 4C, S5B-C). Thus, Shot is required to recruit Patronin to the fusome, thereby transmitting fusome asymmetry to Patronin localization.

The MT-dependent enrichment of Patronin in one cell as the cyst moves through the germarium suggests its initial, weakly asymmetric distribution on the fusome is then amplified by Dynein-dependent transport towards the minus ends of the MT that have been stabilized by Patronin. We tested Dynein function by examining components of the Dynein/dynactin complex that are required for oocyte specification: *egl*, *BicD* and *Arp1* (22–24), (Fig. 4D, S6A-6B). Like MT depolymerization, mutations in any of these genes disrupt the enrichment of Patronin foci in one cell. Deletion of the MT minus end-binding domain of Patronin, but not the CKK domain (25), also prevents Patronin accumulation in the pro-oocyte (Fig. S6C-D). Thus, Patronin localization depends on its binding to MT minus ends and on Dynein activity, suggesting that Dynein transports Patronin bound to MT minus ends towards the pro-oocyte.

Our observations lead us to propose a 4-step model of cyst polarization and oocyte selection (Fig. 4E). First, during cyst formation, the asymmetric segregation of the fusome leads to the one cell with more fusome material than the rest. Second, in region 2a, Patronin is recruited to the fusome by Shot. The cell with most fusome therefore contains more Patronin, leading to the stabilization of more MT minus ends in this cell and a weakly polarized MT network. Third, Patronin bound MTs in other cells of the cyst are then transported by Dynein along these MTs towards their minus ends in the pro-oocyte. Fourth, this creates a positive feedback loop: as Dynein transports more Patronin and MTs into the cell with most stabilized MT minus ends, more minus ends become stabilized in this cell, amplifying the MT polarity and leading to enhanced Dynein transport of oocyte determinants into this cell. In this way, the small original asymmetry in the fusome is converted into the highly polarized MT network that concentrates the oocyte determinants in one cell.

Patronin is a member of the conserved CAMSAP family, raising the possibility that the molecular mechanisms of oocyte selection in *Drosophila* could be conserved during the formation of mammalian oocytes. Although fusomes have not been observed in mammalian cysts (26), MT-dependent transport of organelles through intercellular bridges has been shown to play an important role in oocyte differentiation in mice (3).

## Supplementary Material

Supplementary materials

## Figures and Tables

**Fig. 1:**
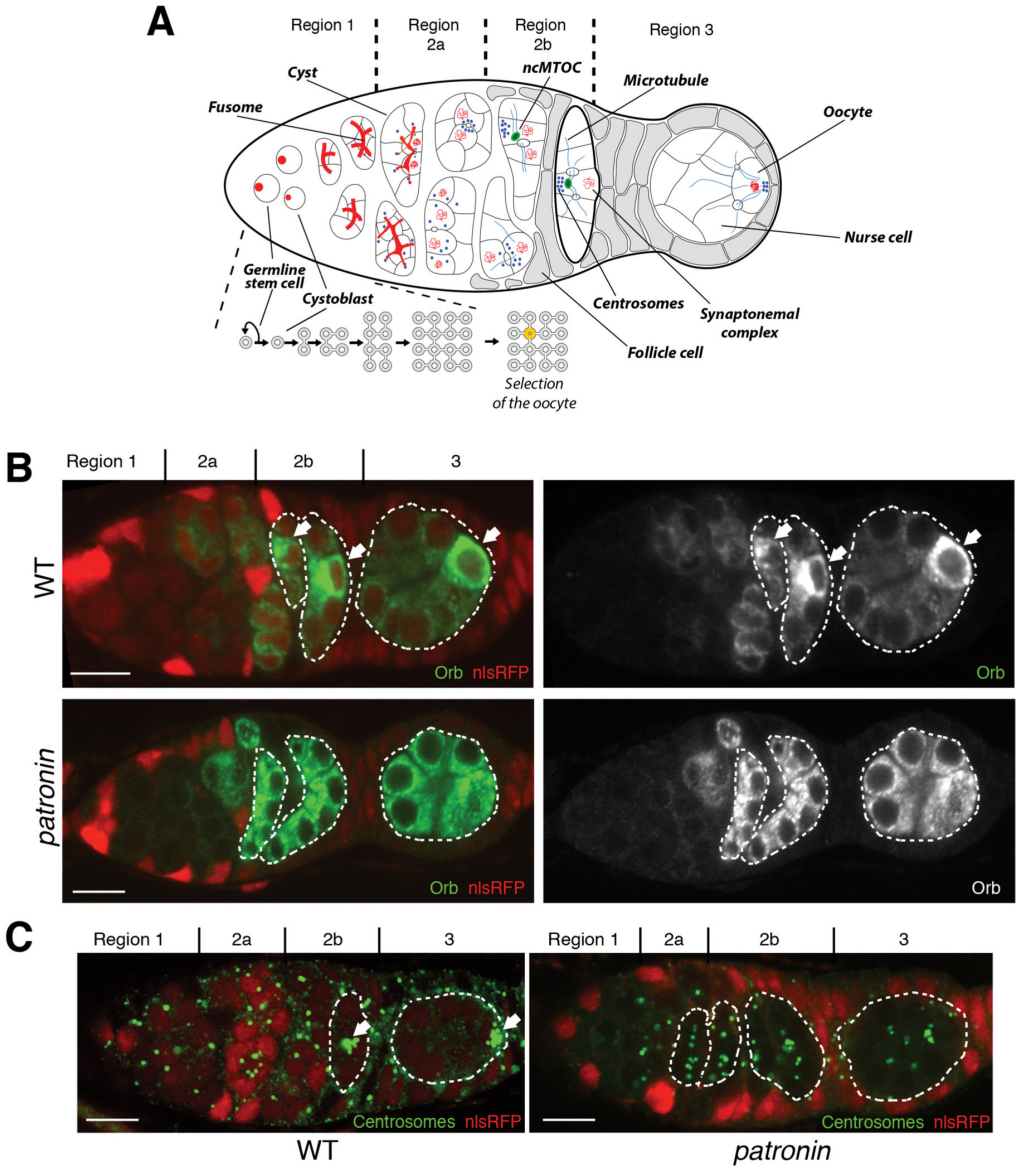
Patronin is required for the oocyte specification. **(A)** A schematic diagram of a *Drosophila* germarium showing germline cyst formation and oocyte selection. Distribution of the oocyte specification markers Orb **(B)** and centrosomes **(C)** in wild type (WT; top or left in C) and *patronin* mutant (bottom or right in C) cysts. For all figures: arrows point to the future oocyte; cysts are marked by dashed lines; mutant cysts are labeled by the absence of nlsRFP; regions of the germarium are indicated on the top; scale bars, 10μm.

**Fig. 2:**
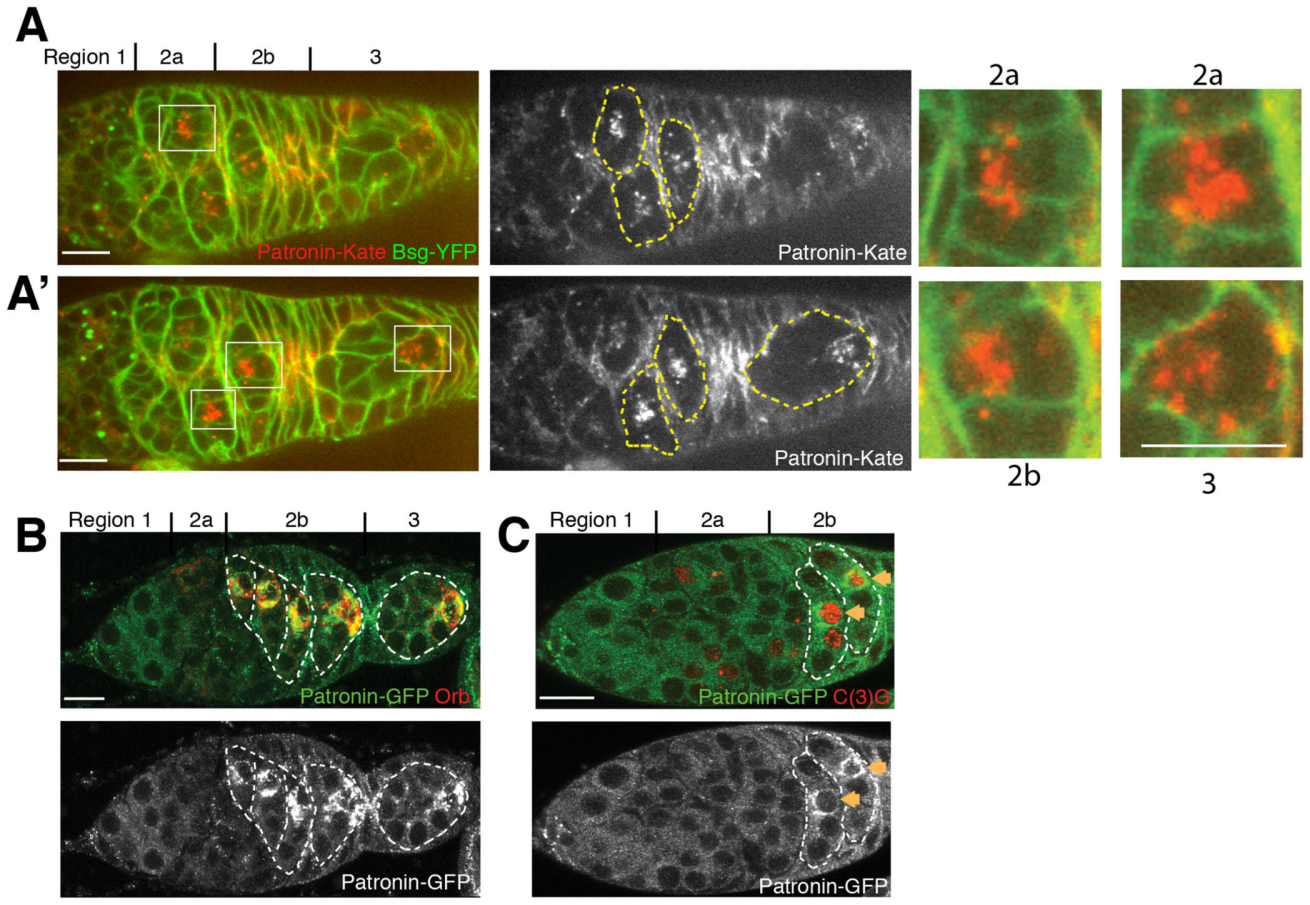
Patronin accumulates in the future oocyte. **(A-A’)** Two different focal planes of a live germarium showing accumulation of endogenously tagged Patronin-Kate in one cell of the cyst. Regions 2a and 2b are shown as close-ups. Cell membranes are labelled by Basigin-YFP (Bsg-YFP). **(B-C)** Ectopically-expressed ubq>Patronin-GFP accumulates in future oocytes labelled by Orb **(B)** or C(3)G **(C)**.

**Fig. 3:**
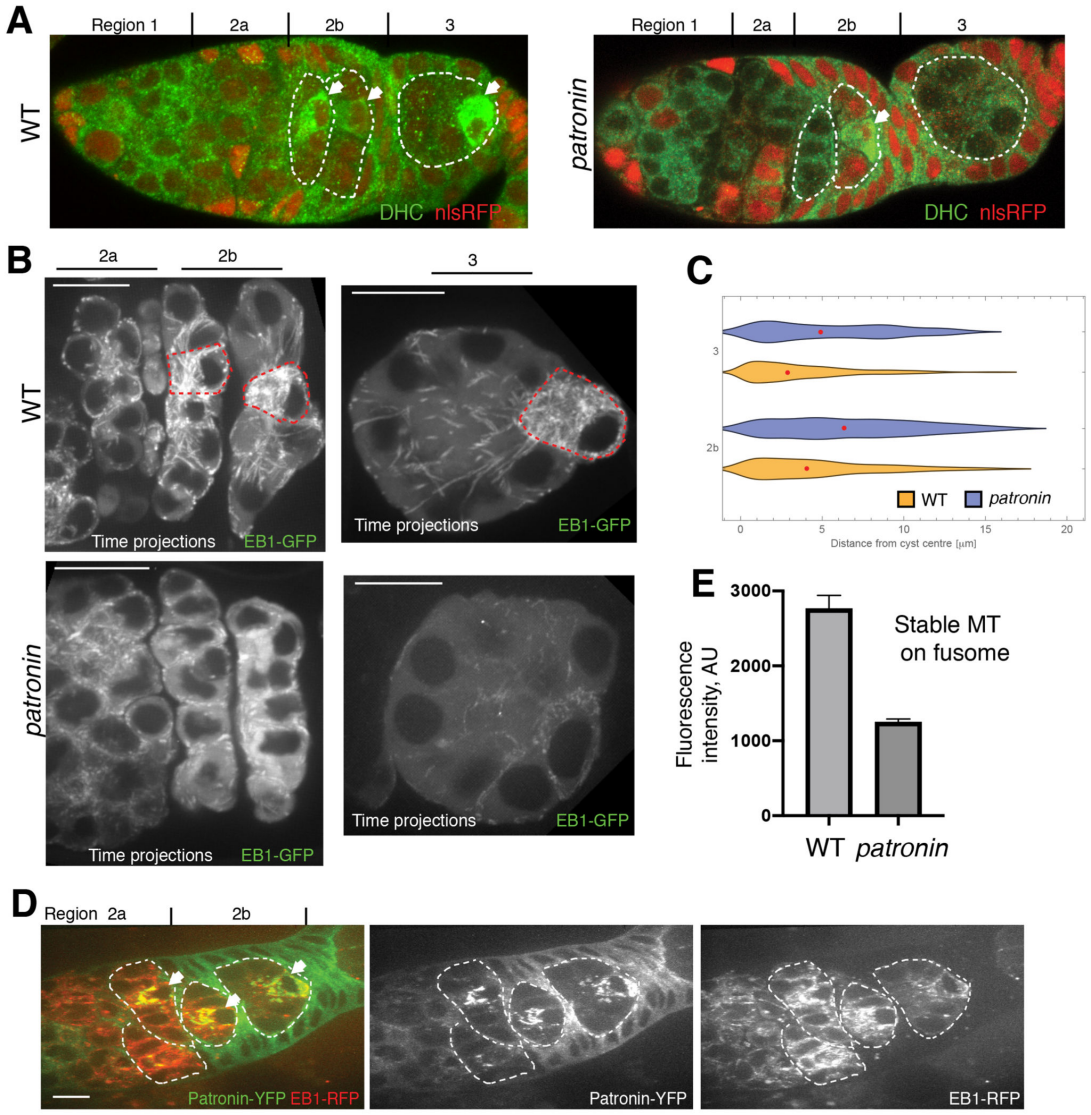
Patronin is required for MT organisation in the cyst. **(A)** Distribution of Dynein Heavy Chain (DHC) in wild type (WT) and *patronin* mutant cysts. **(B-D)** Patronin is required for MTOC formation in the presumptive oocyte. **(B)** EB-1 comet tracks in wild type (WT; top) and *patronin* mutant (bottom) cysts. The images are projections of several time points from Movies S1 (WT; region 2), S2 (WT; region 3), S3 (*patronin*; region 2) and S4 (*patronin*; region 3). The red dashed line marks cells with MTOCs. **(C)** Quantification of EB-1 comet distribution in wild type (WT) and *patronin* mutant cysts in region 3 and 2b of germarium. Red dots indicate median values. **(D)** Live germarium showing co-localization of Patronin-YFP foci with the microtubules plus end marker EB1-GFP in the presumptive oocyte. **(E)** Quantification of the mean fluorescence intensities of fusome associated acetylated microtubules in *patronin* mutant and WT cysts. Errors bars indicate the SEM.

**Fig. 4:**
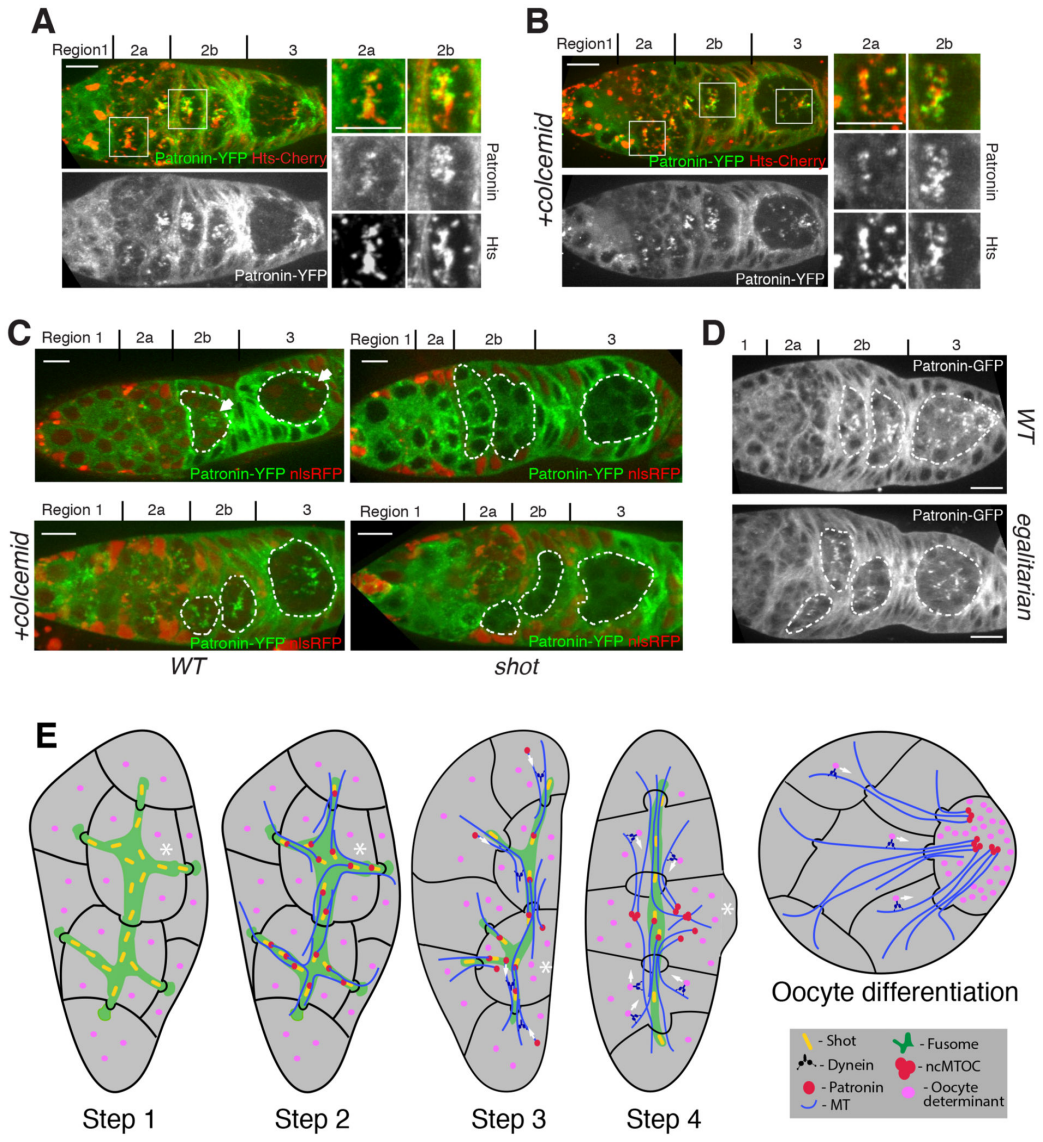
Patronin localisation is defined by fusome and by a positive feed back loop of Dynein mediated transport. **(A-B)** Patronin associates with the fusome in a microtubule-dependent manner. Untreated **(A)** or colcemid-treated **(B)** live germaria expressing Patronin-YFP and Hts-Cherry. Regions 2a and 2b are shown as close-ups. **(C)** Shot links Patronin to the fusome. Live germaria containing wild type (WT; left) and *shot* mutant (right) cysts expressing Patronin-YFP either untreated (top) or treated with colcemid (bottom). **(D)** Patronin localisation depends on Dynein activity. Wild type (WT; top) and *egalitarian* mutant (bottom) live germaria expressing transgenic Patronin-GFP. **(E)** A diagram showing the 4 steps in cyst polarization that lead to the specification of the oocyte and its subsequent positioning at the posterior of the cyst in region 3. See text for details. Asterisk indicates the presumptive oocyte.

## Data Availability

All data are available in the main text or the supplementary materials.

## One-Sentence Summary

Patronin and Dynein form a positive feedback loop that amplifies a weak fusome asymmetry to specify the *Drosophila* oocyte.
