# Supplementary material for: Symmetry breaking in the female germline cyst
Source: Science. Author manuscript; Available in PMC 2021 Nov 19. (PMC7612008; doi:10.1126/science.abj3125)
Supplement: Supplementary materials [file EMS138671-supplement-Supplementary_Materials.pdf]

Science AAAS

Supplementary Materials for

Symmetry breaking in the female germline cyst

D. Nashchekin, L. Busby, M. Jakobs, I. Squires and D. St Johnston

Correspondence to: : d.stjohnston@gurdon.cam.ac.uk, d.nashchekin@gurdon.cam.ac.uk

This PDF file includes:

Materials and Methods
Supplementary text
Figs. S1 to S6
Captions for Movies S1 to S6
References

Other Supplementary Materials for this manuscript include the following:

Movies S1 to S6

Materials and Methods

Mutant alleles. The following *Drosophila melanogaster* mutant alleles have been described previously and can be found on FlyBase.org: *shot*^{3} (*27*), *BicD*^{r5} (*28*), *arp1*^{c04425} (*29*), *egalitarian*^{1} *and egalitarian*^{2} (*30*). The *patronin*$^{c9\text{-}c5}$ allele was generated by injecting nos::Cas9 embryos (*31*) with a sgRNA targeting the second protein coding exon in *patronin* (5' GGACAT**GC**CCATTACC**G**AAA 3'). *patronin*$^{c9\text{-}c5}$ has a GC (highlighted in bold in the sgRNA sequence) to TA substitution changing Met Pro to Ile Ser; and a deletion of a G (highlighted in bold in sgRNA sequence) changing Glu Thr Val Leu to Lys Arg Tyr STOP, creating a premature stop codon after 105 amino acids. *patronin*$^{c9\text{-}c5}$ is homozygous lethal. The mutant phenotypes of *patronin* $^{c9\text{-}c5}$ were rescued by ubq>Patronin-GFP transgene.

Fluorescent marker stocks. Hts-Cherry was derived by N. Lowe from the Hts-GFP CPTI protein trap line using P-element exchange (*32*, *33*). Basigin-YFP is Cambridge Protein Trap Insertion line (*32*). The following stocks have been described previously: Asterless-Cherry (*34*), UAS EB1-GFP (*35*), Patronin-YFP (*13*), pUbq-Patronin-GFP (*36*) (A isoform). Patronin-mKate, UAS EB1-RFP, pUbq-Patronin-GFP (I isoform), pUbq-PatroninΔMTD-GFP, pUbq-PatroninΔCKK-GFP were from this study.

***Drosophila* genetics.** Germline clones of *patronin*$^{c9\text{-}c5}$, *shot*^{3}*, BicD*^{5}*, arp1*^{c04425} were induced by incubating larvae at 37° for two hours per day over a period of three days. Clones were generated with FRT G13 nlsRFP, FRT 40A nlsRFP, FRT 82B nlsRFP (Bloomington Stock Center) using the heat shock Flp/FRT system (*37*). Germline expression of UAS EB1-GFP and UAS EB1-RFP was induced by nanos-Gal4.

Molecular Biology. The Patronin C-terminal mKate knockin was made by injecting nos::Cas9 embryos (*31*) with a single guide RNA targeting the region of the stop codon in *patronin* (5'-GGCGCTTGTAATCTAAGCGG-3') and a donor plasmid with 4-kb homology arms surrounding the mKate sequence. Patronin-mKate is homozygous viable. A full-length *patronin* RI cDNA was amplified from pUASP mCherry-Patronin (*13*) and cloned together with EGFP into pUbq-attb vector downstream of the polyubiqutin promoter. pUbq-PatroninΔMTD-GFP and pUbq-PatrioninΔCKK-GFP were generated by PCR amplifying the

corresponding fragments (see Ref (25) for details) from pUbq-Patronin-GFP and cloning them into pUbq-attb. EB1 cDNA was amplified from pUASP EB1-GFP and cloned together with tagRFP into pUASP-attb to generate UAS EB1-RFP.

Immunohistochemistry. Ovaries were fixed for 20 min in 4% paraformaldehyde and 0.2% Tween in PBS. Ovaries were then blocked with 1% BSA in PBS for 1 hr at room temperature. Ovaries were incubated with the primary antibody for 16 hr with 0.1% BSA in PBS with 0.2% Tween at 4C and for 4 hr with the secondary antibody at room temperature. We used the following primary antibodies: mouse anti-acetylated tubulin at 1:250 (Sigma); mouse anti-Dynein heavy chain at 1:50 (DSHB Hybridoma Product 2C11-2. Deposited to the DSHB by Scholey, JM); guinea pig anti-Shot (*13*) at 1:500, rabbit anti-dPLP at 1:1000 (gift from J. Raff, University of Oxford, UK), mouse anti-C(3)G at 1:500 (gift from R.S. Hawley, Stowers Institute, US), mouse anti-Orb at 1:10 (DSHB Hybridoma Products 4H8 and 6H4. Deposited to the DSHB by Schedl, P), mouse anti- α Spectrin at 1:200 (DSHB Hybridoma Product 3A9. Deposited to the DSHB by Branton, D. / Dubreuil, R.) Conjugated secondary antibodies (Jackson Immunoresearch) were used at 1:100. In situ hybridizations were performed as previously described (*24*) .

Colcemid Treatment. Flies were starved for 2 hr and then fed colcemid (Sigma) in yeast paste (66 μg/ml) for 17 hr. Ovaries were dissected and imaged as described below.

Imaging. For live imaging, ovaries were dissected and imaged in Voltalef oil 10S (VWR International) on an Olympus IX81 inverted microscope with a Yokogawa CSU22 spinning disk confocal imaging system (60x/ 1.35 NA Oil UPlanSApo and 100x/ 1.3 NA Oil UPlanSApo) or on Leica SP5 confocal microscope (63x/1.4 HCX PL Apo CS Oil). To label cell membranes, ovaries were dissected in Schneider's medium (Sigma) with 10 μg/ml insulin (Sigma) and CellMask (1:2000, Life Technologies), incubated for 10 min at room temperature, washed and transferred to Voltalef oil for imaging. Fixed preparations were imaged using an Olympus IX81 (60x/ 1.35 NA Oil UPlanSApo) or a Leica SP8 (63x/1.4 HC PL Apo CS Oil) confocal microscope. Images were collected with Olympus Fluoview, MetaMorph and Leica LAS AF software and processed using ImageJ. Germaria were imaged by collecting 10–15 z sections spaced 0.5 μm apart. The images in Fig. 2B, Fig. 3A,

Fig. 4A, Fig. 4B, Fig. 4C (bottom panels) and Fig. 4D are projections of several z sections.

EB1 tracking. EB1 comets were tracked using the ImageJ plugin TrackMate. For each movie, tracking performance was visually inspected and optimal tracking parameters chosen accordingly. To determine the distribution of EB1 comets in the cyst we calculated the distance from each EB1 track starting point to the cyst centre. The following number of comets were analysed: WT region 3: 910 comets, WT region 2b: 430 comets; *patronin* mutant region 3: 989 comets, *patronin* mutant region 2b: 1736 comets.

Statistical analyses. The chi-square test was used to test whether values were significantly different between WT and *patronin* mutant cysts. The Mann-Whitney t-test was used to determine significance when comparing fluorescence intensities of acetylated tubulin staining and when measuring the co-localisation of fusome and Patronin. We used the MATLAB implementation of the Kruskal Wallis Test followed by a Tukey post hoc test to determine statistical differences in EB-1 comet distributions. A level of $p < 0.01$ was considered to be statistically significant. No statistical methods were used to predetermine sample size, the experiments were not randomized, and the investigators were not blinded to allocation during experiments and outcome assessment.

Reproducibility of experiments. Images are representative examples from at least three independent repeats for each experiment. The number of cysts analyzed for each experiment were as follows: Fig. 1B (WT 27, *patronin* 27), Fig, 1C (WT 30, *patronin* 41), Fig. S1 (WT 33, *patronin* 31), Fig. 2A (17), Fig. 2B (53), Fig. 2C (32), Fig. 3A (WT 19, *patronin* 14), Fig. 3B (WT 30, *patronin* 29), Fig. 3D (41), Fig. S3 (WT 40, *patronin* 45), Fig. 3E (WT 18, *patronin* 6), Fig. 4A (30), Fig. 4B (54), Fig. 4C (WT 35, WT + colcemid 37, *shot* 28, *shot* + colcemid 31), Fig. S5B (15), Fig. S5C (26), Fig. 4D (WT 40, *egalitarian* 34).

Supplementary text

Author contributions:
Conceptualization: DN, DStJ
Methodology: DN, MJ
Investigation: DN, LB, MJ, IS, DStJ

Visualization: DN, MJ, DStJ

Funding acquisition: DN, DStJ

Project administration: DN, DStJ

Supervision: DN, DStJ

Writing – original draft: DN, DStJ

Writing – review & editing: DN, LB, MJ, IS, DStJ

Competing interests: Authors declare that they have no competing interests.

Data and materials availability: All data are available in the main text or the supplementary materials.

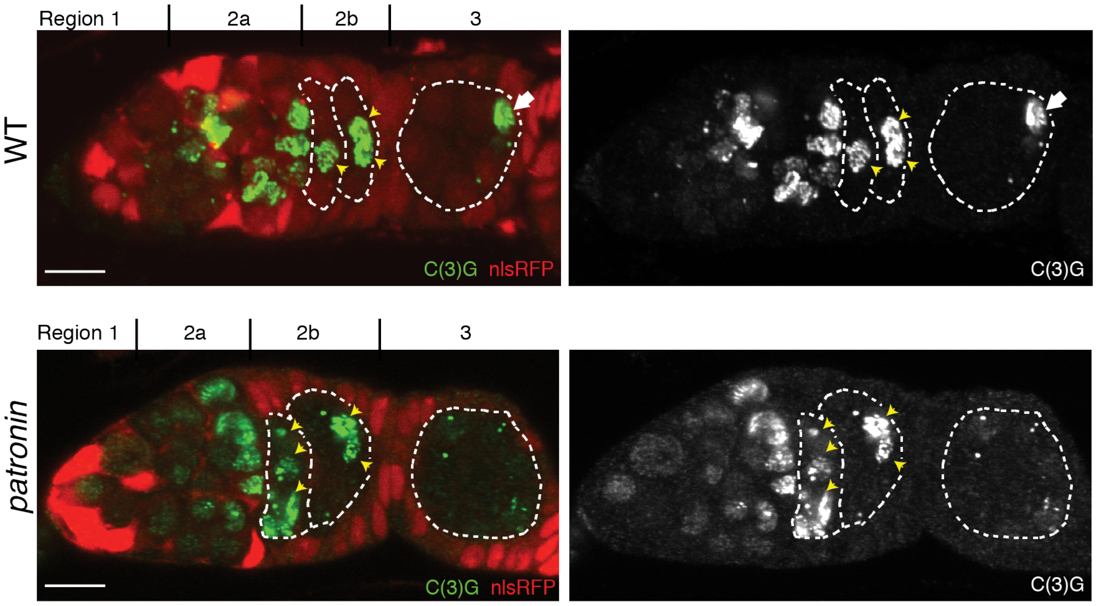

Fig. S1. Patronin is required for the oocyte specification. Distribution of oocyte specification marker C(3)G in wild type (WT; top) and *patronin* mutant (bottom) cysts. Arrows point to the future oocyte. Cysts are marked by dashed lines. Mutant cysts are labeled by the absence of nlsRFP. Arrowheads indicate cells accumulating C(3)G. Regions of the germarium are indicated on the top. Scale bars, 10µm.

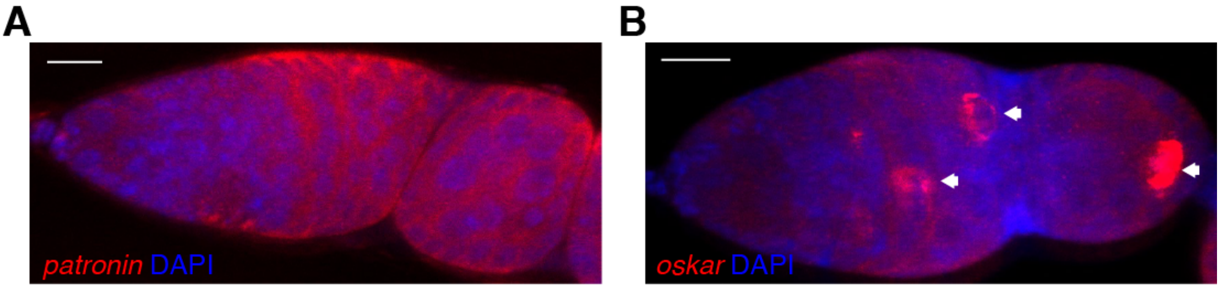

Fig. S2. Patronin mRNA is not localised in the cyst. Confocal images of fluorescent in situ hybridisations (FISH) to endogenous *patronin* **(A)** and *oskar* **(B)** mRNA in a wild type germarium, counterstained with DAPI to label the nuclei. Arrows point to the future oocyte. Scale bars, 10µm.

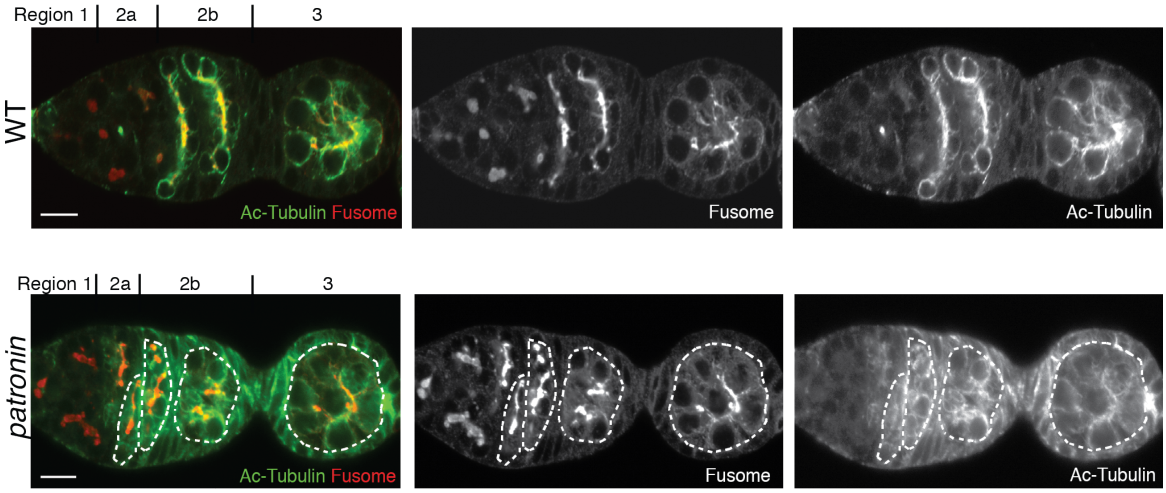

Fig. S3. Patronin stabilises microtubules in the cyst. Wild type (WT; top) and *patronin* mutant (bottom) cysts stained with anti-acetylated tubulin (Ac-Tubulin) and anti-Shot (Fusome). Mutant cysts are marked by dashed lines. Regions of the germarium are indicated on the top. Scale bars, 10μm.

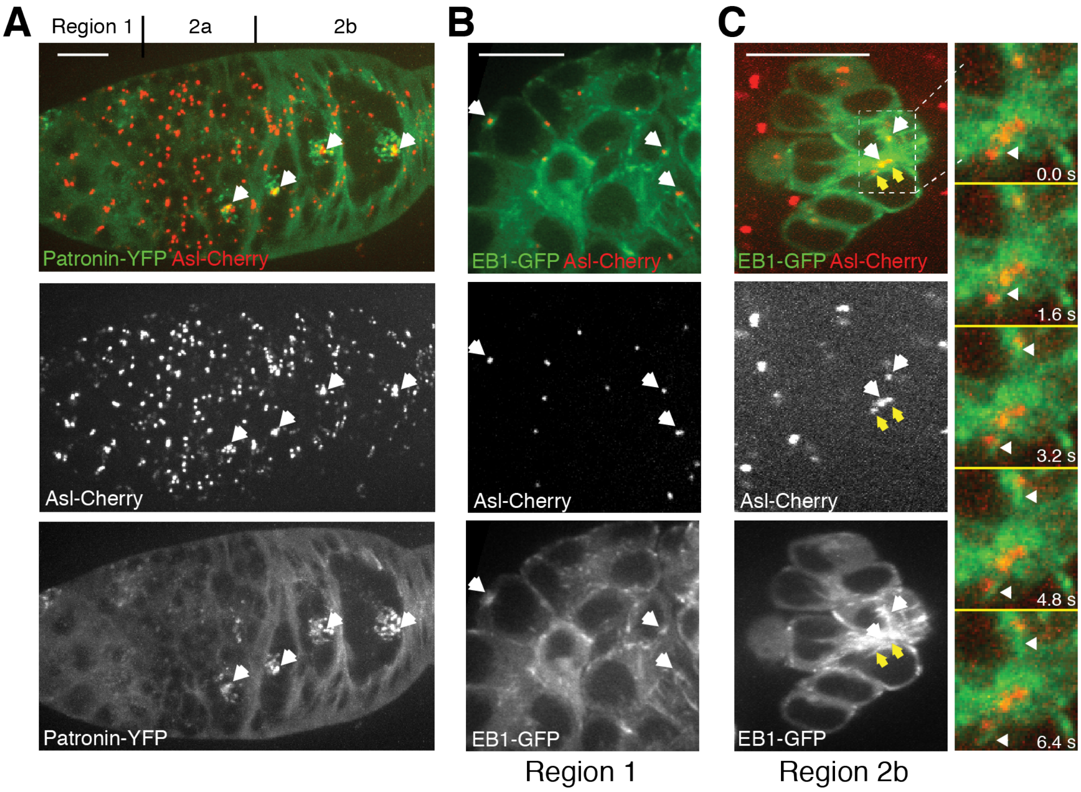

Fig. S4. Patronin MTOCs are not centrosomal. (A) Patronin foci lie outside the centrosomal cluster. Live germarium expressing Patronin-YFP and a centrosomal protein Asterless-Cherry (Asl-Cherry). The image is a projection of several z sections spanning the cyst. Arrows mark centrosomal clusters. **(B-D)** Centrosomes contribute to microtubule organisation in the cyst. Live germaria expressing EB1-GFP and Asterless-Cherry (Asl-Cherry). **(B)** Region 1 of the germarium. The image is taken from Movie S5. Arrows indicate active centrosomes. **(C)** Region 2b of the germarium. The images are projections of several time points from Movie S6. White arrows point to two active centrosomes in the presumptive oocyte. Yellow arrows point to two inactive centrosomes. Close-ups are still images from Movie S6. Arrowheads show new EB1-GFP comets emanating from the active centrosomes. Scale bars, 10μm.

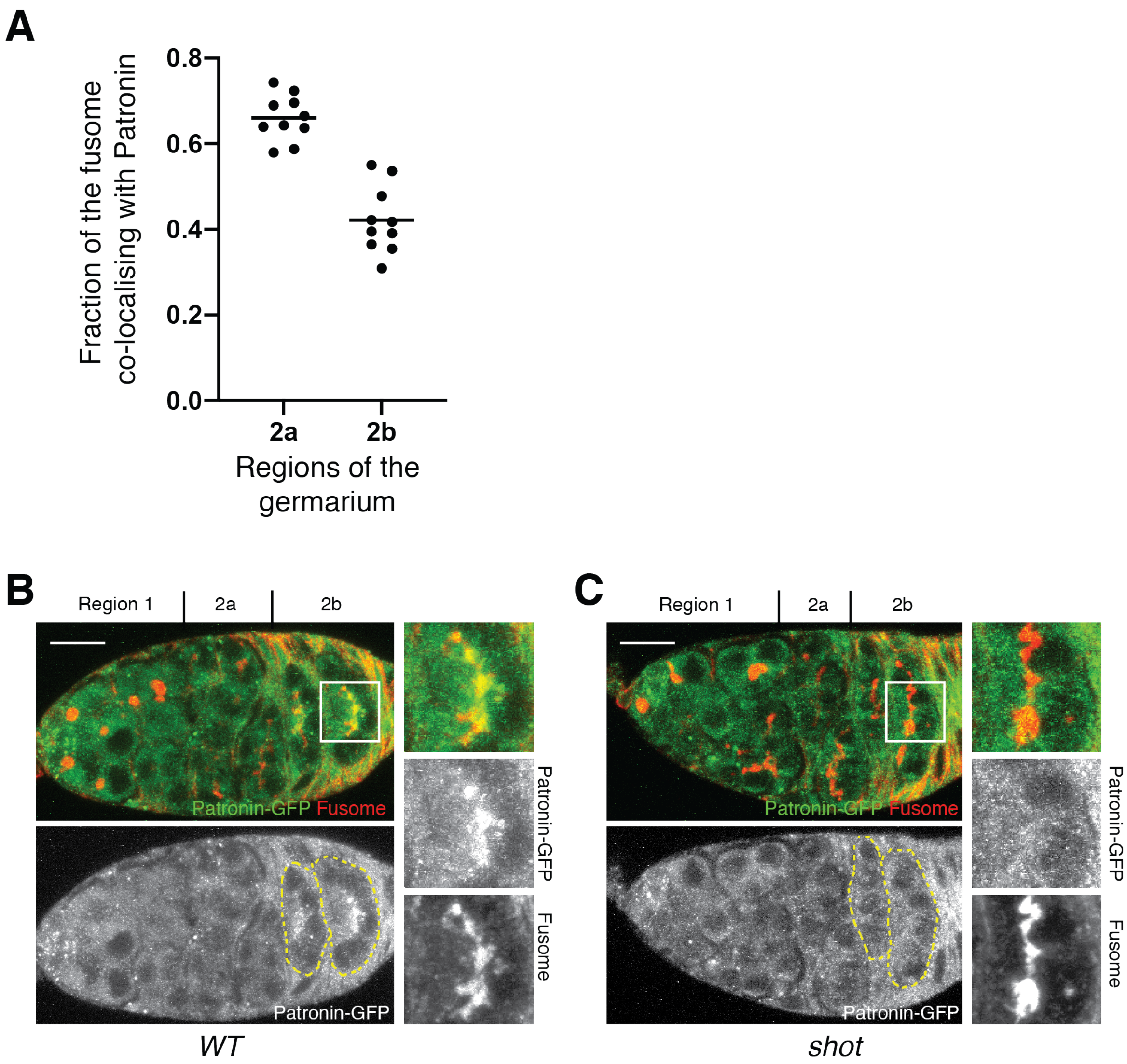

Fig. S5. Patronin associates with the fusome. **(A)** Quantification of fusome and Patronin co-localisation in live germaria expressing Patronin-YFP and the fusome marker Hts-Cherry. Manders' co-localisation coefficient is measured using JACoP plug-in for Fiji. **(B-C)** Shot links Patronin to the fusome. Ectopically expressed Patronin-GFP associates with the fusome in wild type **(B)** but not in *shot* mutant **(C)** cysts. Fusome is labelled by anti-α–Spectrin. Region 2b is shown as a close-up. Regions of the germarium are indicated on the top. Dashed lines indicate cysts in region 2b. Scale bars, 10µm.

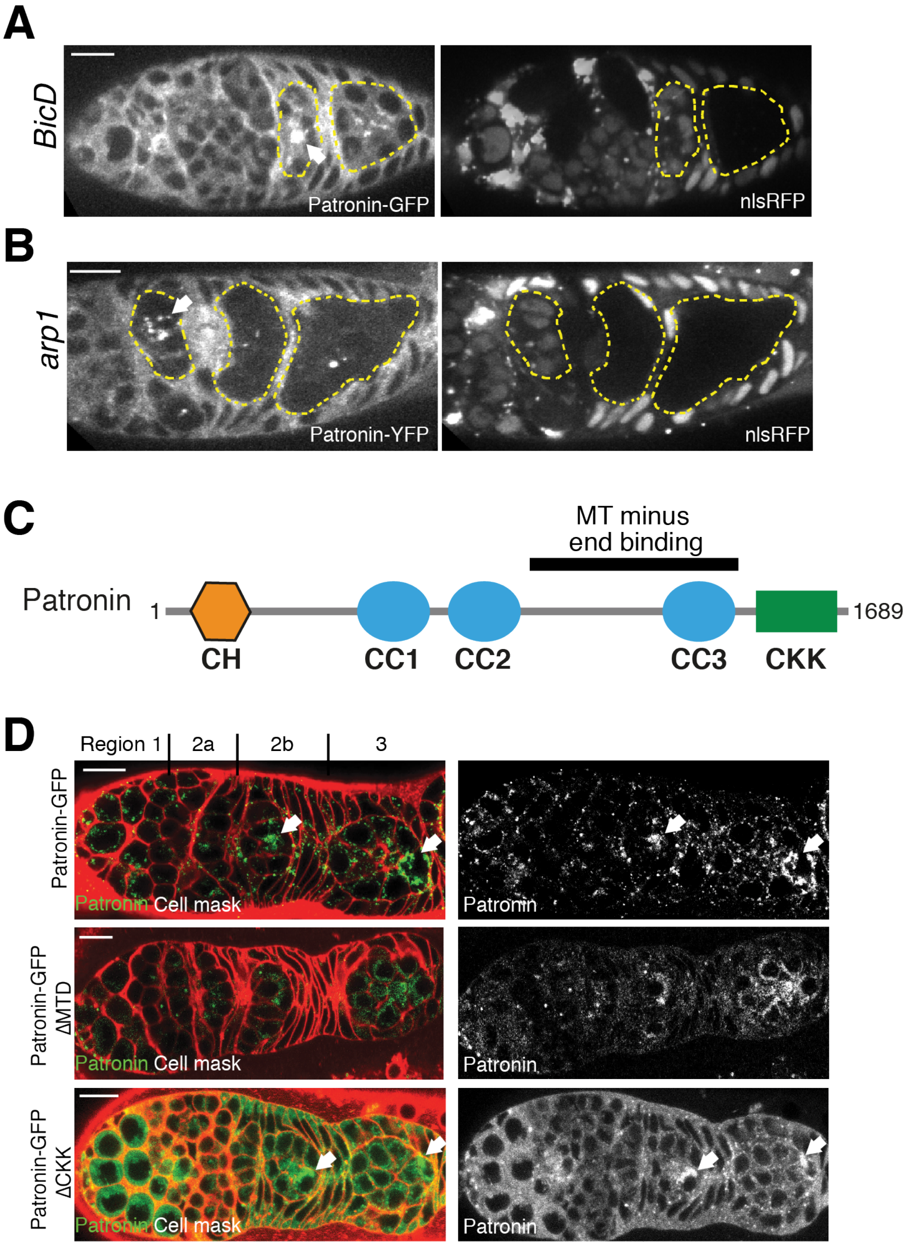

Fig. S6. Patronin localisation depends on Dynein activity and MT minus end binding. (A-B) Live germaria containing *BicD* **(A)** or *arp1* **(B)** mutant cysts expressing transgenic Patronin-GFP **(A)** or Patronin-YFP **(B)**. Cyst are indicated by dashed lines. Mutant cysts are marked by the absence of nlsRFP. Arrows indicate wild type cysts. **(C)** A diagram showing the domain structure of Patronin. **(D)** The MT minus end binding domain (MTD) is required for Patronin localisation. Live germaria expressing wild type (top), MTD-deleted (middle) or CKK-deleted (bottom) transgenic Patronin-GFP. Cell membranes are labelled by CellMask. Arrows indicate accumulation of Patronin foci in the presumptive oocyte. Regions of the germarium are indicated on the top. Scale bars, 10μm.

Movie S1. A time-lapse video of the microtubule plus-end binding protein EB1-GFP in wild-type germline cysts in region 2 of the germaium. The red line outlines the cells containing MTOCs. Related to Figure 3B. Images were collected every 1 second on a spinning disc confocal microscope. The video is shown at 15 frames/sec.

Movie S2. A time-lapse video of the microtubule plus-end binding protein EB1-GFP in wild-type germline cysts in region 3. Related to Figure 3B. Images were collected every 1 second on a spinning disc confocal microscope. The video is shown at 15 frames/sec.

Movie S3. A time-lapse video of the microtubule plus-end binding protein EB1-GFP in *patronin* mutant germline cysts in region 2. Related to Figure 3B. Images were collected every 1 second on a spinning disc confocal microscope. The video is shown at 15 frames/sec.

Movie S4. A time-lapse video of the microtubule plus-end binding protein EB1-GFP in *patronin* mutant germline cysts in region 3. Related to Figure 3B. Images were collected every 1 second on a spinning disc confocal microscope. The video is shown at 15 frames/sec.

Movie S5. A time-lapse video of the microtubule plus-end binding protein EB1-GFP (green) and the centrosomal protein Asterless-Cherry (red) in wild-type germline cysts in region 1. Arrowheads point to active centrosomes. Related to Figure S4B. Images were collected every 1.6 seconds on a spinning disc confocal microscope. The video is shown at 15 frames/sec.

Movie S6. A time-lapse video of the microtubule plus-end binding protein EB1-GFP (green) and centrosomal protein Asterless-Cherry (red) in wild-type germline cysts in region 2b. The white arrow points to an active centrosome in the presumptive oocyte. Related to Figure S4C. Images were collected every 1.6 seconds on a spinning disc confocal microscope. The video is shown at 15 frames/sec.

References and Notes

1. K. Lu, L. Jensen, L. Lei, Y. M. Yamashita, Stay Connected: A Germ Cell Strategy. *Trends Genet*. **33**, 971–978 (2017).

2. L. Lei, A. C. Spradling, Mouse primordial germ cells produce cysts that partially fragment prior to meiosis. *Development*. **140**, 2075–2081 (2013).

3. L. Lei, A. C. Spradling, Mouse oocytes differentiate through organelle enrichment from sister cyst germ cells. *Science*. **352**, 95–99 (2016).

4. M. de Cuevas, M. A. Lilly, A. C. Spradling, Germline cyst formation in Drosophila. *Annu. Rev. Genet.* **31**, 405-28 (1997).

5. J. Huynh, D. StJohnston, The Origin of Asymmetry: Early Polarisation of the Drosophila Germline Cyst and Oocyte. *Current Biology.* **14**, R438-R449 (2004).

6. W. E. Theurkauf, B. M. Alberts, Y.-N. Jan, T. A. Jongens, A central role for microtubules in the differentiation of Drosophila oocytes. *Development*. **118**, 1169–1180 (1993).

7. M. McGrail, T. S. Hays, The microtubule motor cytoplasmic dynein is required for spindle orientation during germline cell divisions and oocyte differentiation in Drosophila. *Development*. **124**, 2409–2419 (1997).

8. J. Bolívar *et al.*, Centrosome migration into the Drosophila oocyte is independent of BicD and egl, and of the organisation of the microtubule cytoskeleton. *Development*. **128**, 1889–1897 (2001).

9. W. Meng, Y. Mushika, T. Ichii, M. Takeichi, Anchorage of microtubule minus ends to adherens junctions regulates epithelial cell-cell contacts. *Cell*. **135**, 948–959 (2008).

10. A. J. Baines *et al.*, The CKK Domain (DUF1781) Binds Microtubules and Defines the CAMSAP/ssp4 Family of Animal Proteins. *Molecular Biology and Evolution*. **26**, 2005–2014 (2009).

11. S. S. Goodwin, R. D. Vale, Patronin Regulates the Microtubule Network by Protecting Microtubule Minus Ends. *Cell*. **143**, 263–274 (2010).

12. N. Tanaka, W. Meng, S. Nagae, M. Takeichi, Nezha/CAMSAP3 and CAMSAP2 cooperate in epithelial-specific organization of noncentrosomal microtubules. *Proc Natl Acad Sci USA*. **109**, 20029–20034 (2012).

13. D. Nashchekin, A. R. Fernandes, D. St Johnston, Patronin/Shot Cortical Foci Assemble the Noncentrosomal Microtubule Array that Specifies the Drosophila Anterior-Posterior Axis. *Dev Cell*. **38**, 61–72 (2016).

14. V. Lantz, J. S. Chang, J. I. Horabin, D. Bopp, P. Schedl, The Drosophila orb RNA-

binding protein is required for the formation of the egg chamber and establishment of polarity. *Genes Dev*. **8**, 598–613 (1994).

15. A. P. Mahowald, J. M. Strassheim, Intercellular migration of centrioles in the germarium of Drosophila melanogaster. An electron microscopic study. *The Journal of Cell Biology*. **45**, 306–320 (1970).

16. N. C. Grieder, M. de Cuevas, A. C. Spradling, The fusome organizes the microtubule network during oocyte differentiation in Drosophila. *Development*. **127**, 4253–4264 (2000).

17. K. Roper, N. H. Brown, A spectraplakin is enriched on the fusome and organizes microtubules during oocyte specification in Drosophila. *Curr Biol*. **14**, 99–110 (2004).

18. H. Lin, L. Yue, A. C. Spradling, The Drosophila fusome, a germline-specific organelle, contains membrane skeletal proteins and functions in cyst formation. *Development*. **120**, 947–956 (1994).

19. M. De Cuevas, A. C. Spradling, Morphogenesis of the Drosophila fusome and its implications for oocyte specification. *Development*. **125**, 2781–2789 (1998).

20. K. Jiang *et al.*, Microtubule minus-end stabilization by polymerization-driven CAMSAP deposition. *Dev Cell*. **28**, 295–309 (2014).

21. H. Lin, A. C. Spradling, Fusome asymmetry and oocyte determination in Drosophila. *Developmental Genetics*. **16**, 6–12 (1995).

22. C. Navarro, H. Puthalakath, J. M. Adams, A. Strasser, R. Lehmann, Egalitarian binds dynein light chain to establish oocyte polarity and maintain oocyte fate. *Nat Cell Biol*. **6**, 427–435 (2004).

23. B. Suter, R. Steward, Requirement for phosphorylation and localization of the Bicaudal-D protein in Drosophila oocyte differentiation. *Cell*. **67**, 917–926 (1991).

24. R. Nieuwburg *et al.*, Localised dynactin protects growing microtubules to deliver oskar mRNA to the posterior cortex of the Drosophila oocyte. *Elife*. **6**, e27237 (2017).

25. M. C. Hendershott, R. D. Vale, Regulation of microtubule minus-end dynamics by CAMSAPs and Patronin. *Proceedings of the National Academy of Sciences*. **111**, 5860–5865 (2014).

26. M. E. Pepling, A. C. Spradling, Female mouse germ cells form synchronously dividing cysts. *Development*. **125**, 3323–3328 (1998).

27. K. Roper, N. H. Brown, Maintaining epithelial integrity: a function for gigantic spectraplakin isoforms in adherens junctions. *Journal of Cell Biology*. **162**, 1305–1315 (2003).

28. B. Ran, R. Bopp, B. Suter, Null alleles reveal novel requirements for Bic-D during Drosophila oogenesis and zygotic development. *Development*. **120**, 1233–1242 (1994).

29. S. T. Thibault *et al.*, A complementary transposon tool kit for Drosophila melanogaster using P and piggyBac. *Nature Genetics*. **36**, 283–287 (2004).

30. T. Schupbach, E. Wieschaus, Female sterile mutations on the second chromosome of Drosophila melanogaster. II. Mutations blocking oogenesis or altering egg morphology. *Genetics*. **129**, 1119–1136 (1991).

31. F. Port, H.-M. Chen, T. Lee, S. L. Bullock, Optimized CRISPR/Cas tools for efficient germline and somatic genome engineering in Drosophila. *Proceedings of the National Academy of Sciences*. **111**, E2967–76 (2014).

32. N. Lowe *et al.*, Analysis of the expression patterns, subcellular localisations and interaction partners of Drosophila proteins using a pigP protein trap library. *Development*. **141**, 3994–4005 (2014).

33. G. B. Gloor, N. A. Nassif, D. M. Johnson-Schlitz, C. R. Preston, W. R. Engels, Targeted gene replacement in Drosophila via P element-induced gap repair. *Science*. **253**, 1110–1117 (1991).

34. P. T. Conduit, A. Wainman, Z. A. Novak, T. T. Weil, J. W. Raff, Re-examining the role of Drosophila Sas-4 in centrosome assembly using two-colour-3D-SIM FRAP. *Elife*. **4** (2015), doi:10.7554/eLife.08483.

35. T. Zhao, O. S. Graham, A. Raposo, D. St Johnston, Growing microtubules push the oocyte nucleus to polarize the Drosophila dorsal-ventral axis. *Science*. **336**, 999–1003 (2012).

36. H. Wang, I. Brust-Mascher, G. Civelekoglu-Scholey, J. M. Scholey, Patronin mediates a switch from kinesin-13-dependent poleward flux to anaphase B spindle elongation. *The Journal of Cell Biology*. **4**, 1343 (2013).

37. T. B. Chou, N. Perrimon, Use of a yeast site-specific recombinase to produce female germline chimeras in Drosophila. *Genetics*. **131**, 643–653 (1992).
